# A NIRS-Based Technique for Monitoring Brain Tissue Oxygenation in Stroke Patients

**DOI:** 10.3390/s24248175

**Published:** 2024-12-21

**Authors:** Josefina Gutierrez-Martinez, Gabriel Vega-Martinez, Cinthya Lourdes Toledo-Peral, Jorge Airy Mercado-Gutierrez, Jimena Quinzaños-Fresnedo

**Affiliations:** 1 Division for Research in Medical Engineering, Instituto Nacional de Rehabilitacion Luis Guillermo Ibarra Ibarra, Mexico City 14389, Mexico; jgutierrez@inr.gob.mx (J.G.-M.); cltoledo@inr.gob.mx (C.L.T.-P.); jmercado@inr.gob.mx (J.A.M.-G.); 2 Division of Neurological Rehabilitiation, Instituto Nacional de Rehabilitacion Luis Guillermo Ibarra Ibarra, Mexico City 14389, Mexico; jquinzanos@inr.gob.mx

**Keywords:** deoxyhemoglobin, fNIRS, oxyhemoglobin, reflected-light, rehabilitation

## Abstract

Stroke is a global health issue caused by reduced blood flow to the brain, which leads to severe motor disabilities. Measuring oxygen levels in the brain tissue is crucial for understanding the severity and evolution of stroke. While CT or fMRI scans are preferred for confirming a stroke due to their high sensitivity, Near-Infrared Spectroscopy (NIRS)-based systems could be an alternative for monitoring stroke evolution. This study explores the potential of fNIRS signals to assess brain tissue in chronic stroke patients along with rehabilitation therapy. To study the feasibility of this proposal, ten healthy subjects and three stroke patients participated. For signal acquisition, two NIRS sensors were placed on the forehead of the subjects, who were asked to remain in a resting state for 5 min, followed by a 30 s motor task for each hand, which consists of opening and closing the hand at a steady pace, with a 1 min rest period in between. Acomplete protocol for placing sensors and a signal processing algorithm are proposed. In healthy subjects, a measurable change in oxygen saturation was found, with statistically significant differences (females *p* = 0.016, males *p* = 0.005) between the resting-state and the hand movement conditions. This work showed the feasibility of the complete proposal, including the NIRS sensor, the placement, the tasks protocol, and signal processing, for monitoring the state of the brain tissue cerebral oxygenation in stroke patients undergoing rehabilitation therapy. Thus this is a non-invasive barin assessment test based on fNIRS with the potential to be implemented in non-controlled clinical environments.

## 1. Introduction

The effects of a stroke can vary depending on its severity and the location of the injury. These effects can include the loss of sensory sensitivity, paresthesia, vision problems, balance and coordination issues, and language difficulties such as aphasia and apraxia. Additionally, some individuals may experience dysphagia. While the incidence of post-stroke has decreased, the recurrence rate of stroke can range from 5.7% to 51.3% after a first-ever stroke [1]. Other studies have reported recurrence rates of 5.4% [2], 7–20% at 1 year, and 16–35% at 5 years [3].

The pathophysiology of stroke involves a reduction in blood flow to nervous tissue, either due to a blocked blood vessel or a bleed. This reduction in blood flow leads to a decreased supply of glucose and oxygen, resulting in oxidative stress, necrosis, and neuronal cell death [4]. The middle cerebral artery is often the most affected, leading to frequent motor alterations such as hemiplegia and hemiparesis of the upper limb [5]. Thus, stroke causes significant changes in brain tissue oxygenation, affecting both the oxygen saturation and concentrations of oxygenated (HbO_2_) and deoxygenated (HHb) hemoglobin, as well as cerebral blood flow [6].

The recovery of motor or cognitive functions after a stroke requires a long time and resources. Timely treatment and diagnosis should focus on preventing stroke recurrence. To achieve this, effective and accessible monitoring tools are needed to measure changes in brain tissue oxygenation. This can help assess the effects of neurorehabilitation therapy in post-stroke patients to prevent secondary damage to brain tissue, avoid motor sequelae, and even prevent death.

An important parameter in the diagnosis and classification of stroke severity and recurrence is the measurement of the oxygenation status of the neuronal tissue. Positron emission tomography (PET) is the gold standard for determining brain oxygenation [7]. magnetic resonance imaging (MRI) and Computed Tomography (CT) are useful studies to confirm stroke, with CT being the study of choice due to its high sensitivity and specificity. However, CT carries risks due to exposure to X-rays. In addition, PET, MRI, and CT are expensive and complex studies that are not available options for most hospitals.

There is evidence of the application of near-infrared spectroscopy (NIRS) as a non-invasive technique to monitor cerebral oximetry. It is useful in preventing cerebral hypoxemia in both intensive care units and perioperative settings [6], with exemptions such as in cases of brain death diagnosis [8]. A systematic review by Yang et al. suggests that NIRS could monitor function recovery and therapeutic effects and predict the risk in stroke patients [9].

NIRS is a non-invasive, optical, and low-cost technique used to measure the absorption of light at various wavelengths. The low absorption capacity of biological tissue allows optic methods to be used to measure hemodynamic responses [10] and determine tissue oxygenation [11]. NIRS is a widely used non-invasive method to assess cerebral activity due to its linear relationship with hemodynamics. The NIRS signal has been validated with neuroimaging techniques, showing a strong correlation with PET and functional MRI measures [12].

NIRS relies on the principle of absorption spectroscopy, where molecules have a unique absorption spectrum. This allows for identification and quantification based on the wavelengths absorbed, so that the attenuated light level is interpreted as a function of changes in chromophore concentration. It is important to note that with a wavelength over 900 nm, the water absorption factor increases, which can make the measurements of chromophore concentration difficult [13]. By measuring light attenuation at two different wavelengths, 690 nm and 830 nm, data related to the concentrations of the chromophores oxygenated and deoxygenated hemoglobin can be obtained [14,15]. With these data, the arterial hemoglobin oxygen saturation can be measured.

NIL beams can penetrate bones, with photons ranging from 700 to 1100 nm capable of deeper penetration of several centimeters or more. This makes it feasible for transcranial cerebral oximetry measurement [16]. Tissue oxygen saturation is the result of a combination of blood in the arteries (25%), capillaries (5%), and veins (70%). The NIRS estimates the percentage of the concentrations of HbO_2_ in both arterial and venous blood compartments. Therefore, NIRS is a method that can provide continuous and non-invasive monitoring of regional oxygen saturation in the central nervous system.

In the single-distance configuration, the light sources and photodetectors are positioned next to each other. This arrangement enables the calculation of the differential path length factor, which represents the average distance a photon travels through the tissue between the source and detector. This factor is included in a mathematical model based on the modified Beer–Lambert law. It allows for the absolute measurement of tissue oxygenation, presenting relative changes in tissue oxygenation as the tissue saturation index [17].

In this wark, a feasibility pilot study is presented, aimed to characterize and evaluate the performance of a NIRS sensor in detecting changes in brain tissue oxygenation for the monitoring of rehabilitation therapy. An algorithm based on the modified Beer–Lambert law was developed to calculate the concentrations of HbO_2_ and HHb from SpO_2_ using fNIRS signal recordings (red and infrared signals).

## 2. Materials and Methods

The following section provides a detailed description of the algorithm used for calculating the concentrations of HbO_2_ and HHb, as well as SpO_2_. It also covers the population description and the module for recording fNIRS signals. Furthermore, it includes information on the configuration and positioning of the optodes, as well as the general experimentation protocol.

This work uses the definition of the NIRS sensor as the red and infrared light acquisition device, its geometry, and the algorithm that allows the acquisition of a signal. The fNIRS signal is obtained as a response to a functional evaluation of the changes in oxygen saturation and the concentrations of HbO_2_ and HHb.

### 2.1. Population

This study involved 10 healthy adults and 3 stroke patients whose laterality was previously confirmed by MRI studies assessed by an independent radiologist. This analysis allowed the precise identification of the side of the brain affected by the stroke, differentiating it from healthy tissue, which was essential to focus the study on the corresponding brain areas during intervention and analysis. The radiologist was unaware of the NIRS measurements. The inclusion criteria for the stroke patients were as follows: they had hemiparesis, were attending a neurological rehabilitation program, and did not have psychiatric disorders, alcohol use disorders, or drug use disorders. Before the research, all the eligible participants were informed about the purpose of the project and provided their informed written consent to participate per the Declaration of Helsinki. The research was approved by the Research and Ethics Committees of the Instituto Nacional de Rehabilitation LGII (registration number 24/24).

### 2.2. Data Acquisition Module

**Figure 1:**
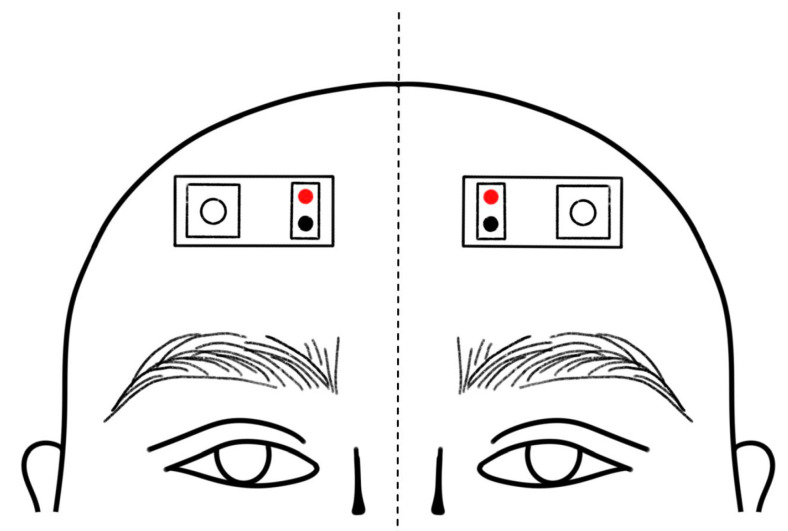
NIRS sensor placement.

The measurement of transcranial cerebral blood oxygen used two NIRS sensors (optodes, functional near-infrared spectroscopy (FNIRS) sensor, Biosignalplux©, PLUX, Lisbon, Portugal) and two different wavelengths: 660 nm for red light (λ1) and 850 nm for infrared light (λ2). These optodes come calibrated by the manufacturer to detect signals in the brain area. Each optode consists of 2 light-emitting diodes, one for red light and the other for infrared light, as shown in Figure 1.

The NIRS sensors were placed on the frontal regions of the scalp (centered by C_z_, according to the 10–20 international system [18]), covering the motor cortex of both the right and left hemispheres, as shown in Figure 1. The sensors were secured tight using a stretch headband horizontally around the head, which ensures that the light does not leak out, and no external light enters. The NIRS signals were acquired with a sample frequency of 1000 Hz.

The amplitude of the current intensity signals of the red and infrared reflected light between the right sensor and the left sensor should be as similar as possible, with a maximum difference of 5%.

### 2.3. Signal Acquisition Protocol

In the case of patients, the experiment protocol goes as follows: start with a 5 min recording to capture the basal signal, then perform 30 s of hand movements on the healthy side, followed by a 1 min rest. After the rest, perform another 30 s of hand movements, this time on the affected side, followed by another 1 min rest. During the hand movements, the patient should comfortably open and close their hand at a manageable pace for the duration of the 30 s, especially on the affected side.

In the case of healthy subjects, start the experiment protocol with a 5 min recording to capture the basal signal, then perform 30 s of hand movements on the dominant side, followed by a 1 min rest. After the rest, perform another 30 s of hand movements, this time on the non-dominant side, followed by another 1 min rest. During the hand movements, the subject should comfortably open and close their hand at a manageable pace for the duration of the 30 s.

### 2.4. Pre-Processing

Once both raw signals (red and ired) had been acquired, the discrete wavelet transform (DWT) was applied to remove the high-frequency components related to environmental noise, biological causes, and mechanical artifacts. DWT is a convolutional operation between the mother wavelet and the signal to be processed. Consequently, the selection of the wavelet used for filtering was based on the artifact waveform; hence, the Daubechies db4 wavelet was selected. The decomposition level was set to 5. The proposed filter uses a Bayesian thresholding method where it is assumed that the wavelet coefficients can be modeled as a combination of two components: useful signal and noise. The assumption is that the coefficients of the useful signal follow a Gaussian distribution with zero mean and a certain variance value, while the coefficients associated with noise are modeled as a Gaussian distribution with zero mean, but with a different variance. Once the threshold is calculated, it is applied to the wavelet coefficients, and those below the threshold are filtered as they are considered the noise part of the signal while those above the threshold are retained.

### 2.5. Mathematical-Model Algorithm

The changes in the waveform of absorbed light signals are often linked to alternating current (AC) and direct current (DC) components, which reflect different physiological aspects. The AC component of the red (red) and infrared (ired) signals is associated with the rhythmic changes in blood volume due to heartbeats, representing the pulsating blood flow. It typically appears as a periodic wave. On the other hand, the DC component is slower and represents non-pulsatile blood volume, the effects of vascular tone, and respiration.

The AC/DC ratio is used to compare the pulsatile change in the signal with the average absorption level and is employed to calculate oxygen saturation (SpO_2_). Additionally, the red/ired absorbance, known as the “*AR*”, is used to calculate the relationship between the intensity of the emitted and received light of the red (λ1) and the infrared (λ2) signals. The steps of the algorithm are described in Table 1.

(1) $$AR=\frac{\left({AC}_{red}/({DC}_{red}\right)}{\left({AC}_{ired}/({DC}_{ired}\right)}$$

Once the signals, red and ired, are conditioned, a mathematical model based on modified Beer–Lambert law is used for calculating the concentrations of HbO_2_ and HHb from the SpO_2_, for each sample of each cardiac cycle.

**Table 1:** Mathematical models and procedures used in the calculation of the concentrations of HbO_2_ and HHb.

Step	Description	How
1. Convert pre-process data into SpO_2_ values for each cardiac cycle	Convert digital values from the raw signal to current values (*I*)	Transfer function (2) $I=\frac{0.15\times ADC}{2^{n}}$ where *I* is the current in [μA], *ADC* is the value sampled from the channel, and *n* is the number of bits of the channel
	Absorbance Ratio (AR) is calculated as a function of VPP and Vavr	- the peaks in both signals (λ1 and (λ2) are first identified - the cardiac cycles are segmented - the peak-to-peak value (VPP) is measured for AC - the average value (Vavr) is calculated for DC
		(3) $$AR=\frac{\left({VPP}_{red})\times ({Vavr}_{ired}\right)}{\left({Vavr}_{red}\right)\times ({VPP}_{ired})}$$
	The percentage of SpO_2_ is calculated	The absorbance ratio is converted by applying the following equation: (4) ${SpO}_{2}\left(\%\right)=A-B\times AR$ where A and B are constants based on the calibration of the NIRS sensor. A= 110 and B = 25
2. Apply the Differential Path Factor (DPF)	Adjust the effective optical path length	(5) $DPF=\frac{L_{ef}}{d}$ where L_ef_ is the effective optical path length, d is the distance between the source and the detector
3. Apply the molar extinction coefficient	Determine how strongly each chromophore absorbs light at each wavelength λ1 and λ2	(6) $\epsilon =\frac{A}{c\cdot l}$ where ε(λ) is the molar extinction coefficient, *c* is the concentration value of each chromophore, and *l* is the optical path length.
4. Calculate HbO_2_ and HHb	Apply the modified Beer–Lambert law to convert absorbance changes ΔA(λ) to concentration changes	(7) ΔA(λ)=Δ(ϵ(λ)⋅c⋅L) where L is the effective optical path length adjusted from DPF
	For biological tissues, L is adjusted using DPF	(8) ΔA(λ)=Δ(ϵ(λ)⋅c⋅DPF⋅d) where ε(λ) is the molar extinction coefficient, *c* is the concentration value of each chromophore, and L is the effective optical path length adjusted from DPF
	The molar extinction coefficient ε is converted to the absorption coefficient α for each λ1 and λ2 and for each chromophore	α_HbO_2__λ1 = 445 × 2.303 α_HHb_λ1 = 3442 × 2.303 α_HbO_2__λ2 = 1097 × 2.303 α_HHb_λ2 = 781 × 2.303
	The system of equations is solved	$\Delta A(\lambda 1)=aHbO_{2}\;(\lambda 1)\cdot \Delta [HbO_{2}]+aHHb(\lambda 1)\cdot \Delta [HHb]$ $\Delta A(\lambda 2)=a{HbO}_{2}(\lambda 2)\cdot \Delta [{HbO}_{2}]+aHHb(\lambda 2)\cdot \Delta [HHb]$

**Figure 2:**
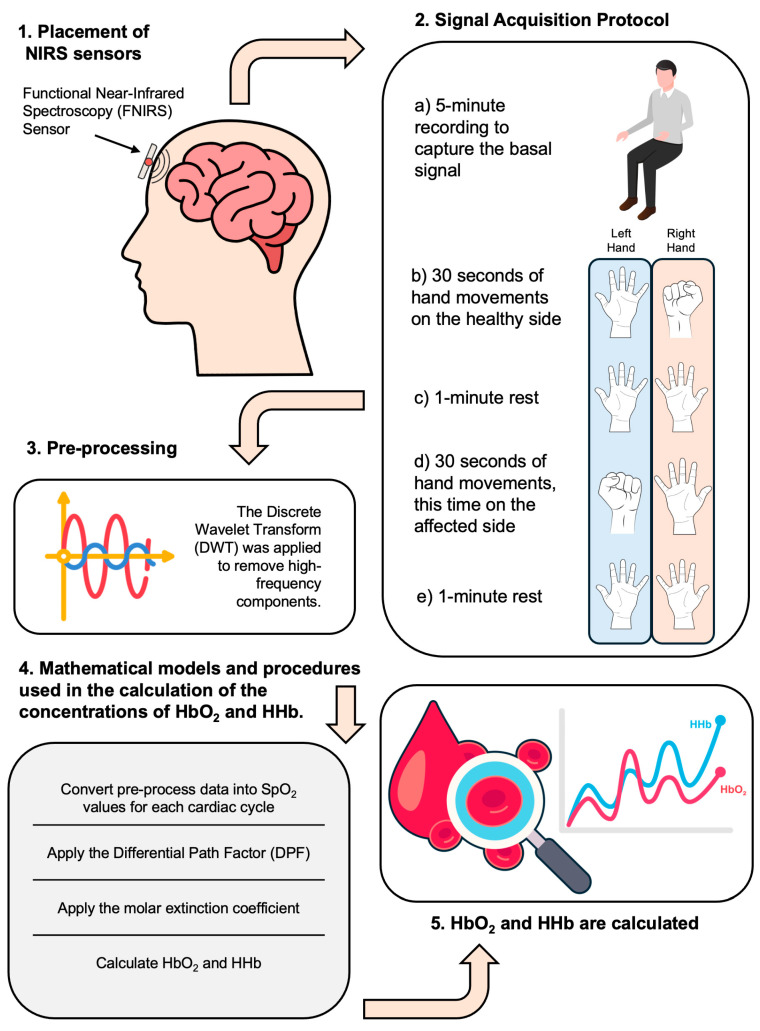
Methodology proposal comprising the NIRS sensors placement, the rest/movement task signal acquisition protocol, and its processing to obtain brain tissue oxyhemoglobin and deoxyhemoglobin concentrations.

The complete methodology proposal comprises the NIRS sensors placement, the rest and movement task protocol for signal acquisition, as well as the signal processing proposed to obtain brain tissue oxyhemoglobin and deoxyhemoglobin concentrations, Figure 2.

### 2.6. Statistical Analysis

The fNIRS signals from healthy subjects were analyzed using descriptive statistics and tested for normality using the Shapiro–Wilk test. Then, the signals from the rests were compared to the signals from the motor tasks using Student’s T test or the Mann–Whitney U test (*p* < 0.05).

## 3. Results

Ten healthy adults, five men and five women, one left-handed, with a mean age of 34.6 years old (range: 22–45 years), were recruited from the community as healthy control subjects. In addition, three stroke patients also participated, and they are described as follows:

- Patient 1—Male, 18 years old, stroke survivor, 5-year evolution at the right brain hemisphere, and left-handed.
- Patient 2—Female, 36 years old, stroke survivor, 5-year evolution at the left brain hemisphere, and right-handed.
- Patient 3—Male, 70 years old, stroke survivor, 1-month evolution at the left brain hemisphere, and right-handed.

These patients attended the institution’s Acquired Brain Injury service for rehabilitation therapy. As stated in the signal acquisition protocol in the methodology section, measurements were taken from two sensors placed in the forehead during rest and while performing motor tasks (opening and closing hand).

The algorithm starts by processing the fNIRS signal records from two channels, left and right, into digital values. Each record is composed of 511,000 samples corresponding to 8 min and 30 s, which is the execution time of the protocol that evaluates rest and movement. The time that the algorithm takes to execute all the steps, described in Table 1, is 89 ± 1.23 s for each record of the 10 healthy subjects and the three patients.

There are not many mechanical artifacts, since the test is performed at rest and the requested movement only consists of opening and closing the hand. The high-frequency artifact presents more frequently due to the location of the NIRS sensor; its elimination is treated in step 1 of Table 1. High frequency increases when the sensor moves towards C_z_ and due to ambient light, and the direct incidence of other light sources in the region where the sensors are used must be avoided.

The calculation of the concentration of oxyhemoglobin and deoxyhemoglobin is more complex, as it is based on matrix operations, so the calculation of oxygen saturation consumes more than 70% of the processing time.

The identification of cardiac cycles is based on the location of peaks, and it operates by thresholds. It is necessary to consider that in the first part of the record, the fNIRS signals are more attenuated and do not allow the correct establishment of the threshold; to overcome this, the first 5 s of each record are eliminated.

Before the calculation of oxygen saturation, it is necessary to validate the synchrony in time between the peaks identified in the reflected red and ired light. The variation between each cardiac cycle in both signals must not be greater than 100 ms, otherwise the cycle must be eliminated.

**Figure 3:**
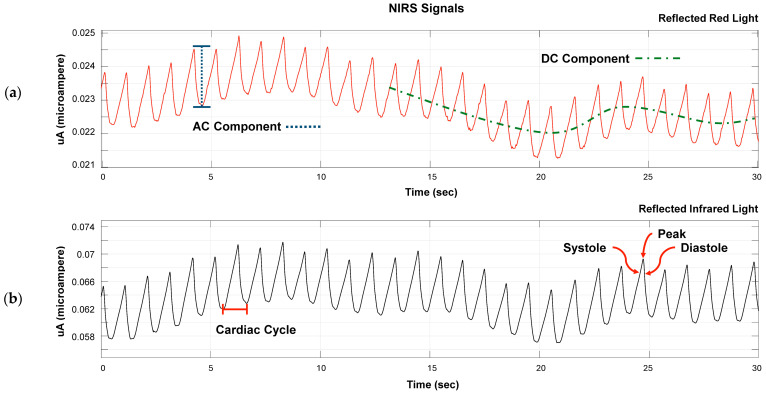
Oxygen saturation signals. The AC and DC components are shown. The segments that represent a heartbeat are also present as systole, diastole, and peak. (**a**) Reflected red light, signal λ1. (**b**) Reflected infrared light, signal λ2.

Figure 3 shows the reflected light corresponding to raw signals from sensors 1 and 2 after the signals were converted from digital values to current intensity values, I (mA), by applying the transfer function described earlier in Equation (2).

In the fNIRS signals, three components can be identified, which are associated with changes in blood volume in the small blood vessels of tissues. The systole represents the upward phase of the wave, indicating an increase in the blood pressure and vessel blood volume. The peak corresponds to the maximum blood volume during a cardiac cycle. Lastly, the diastole is the downward phase of the wave, signifying a decrease in the blood pressure and vessel blood volume.

**Figure 4:**
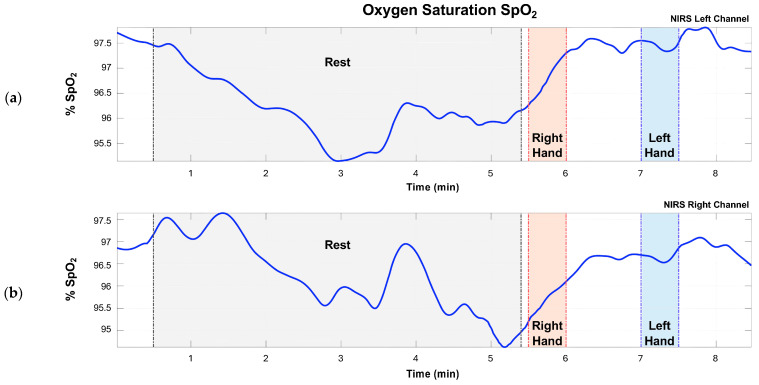
Brain SpO_2_ variation is observed in a healthy subject. The time segments that correspond to rest and right- and left-hand movement can be seen in light gray, orange, and blue, respectively. (**a**) NIRS left channel. (**b**) NIRS right channel.

After obtaining the current intensity values for each signal I (λ1) and I (λ2) and applying the absorbance ratio, Equation (3), the SpO_2_ percentage is calculated using Equation (4). Figure 4 shows the graph of the variation in the cerebral SpO_2_ of a subject for each cardiac cycle.

**Figure 5:**
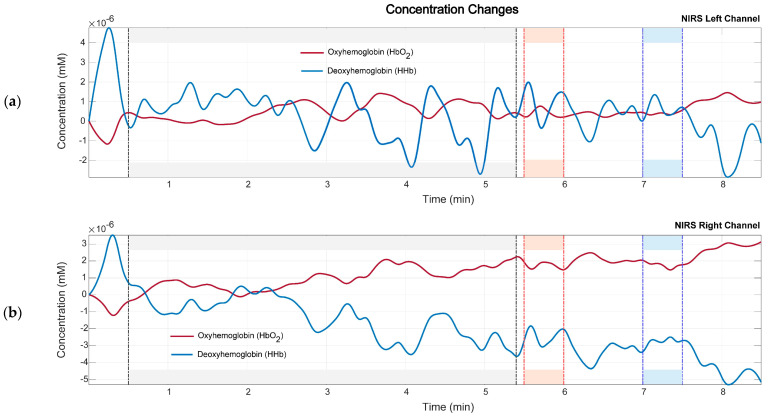
Concentrations of the chromophores for oxygenated (HbO_2_) and deoxygenated (HHb) hemoglobin for a healthy subject. The gray area corresponds to the rest state, the light orange area corresponds to the right-hand movement, and the light blue area corresponds to the left-hand movement. (**a**) NIRS left channel. (**b**) NIRS right channel.

**Figure 6:**
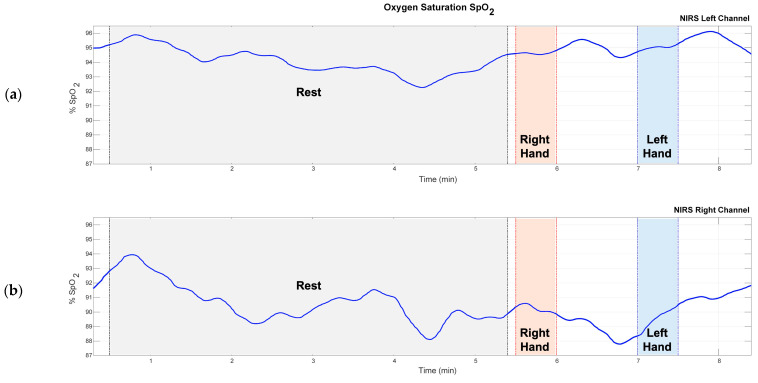

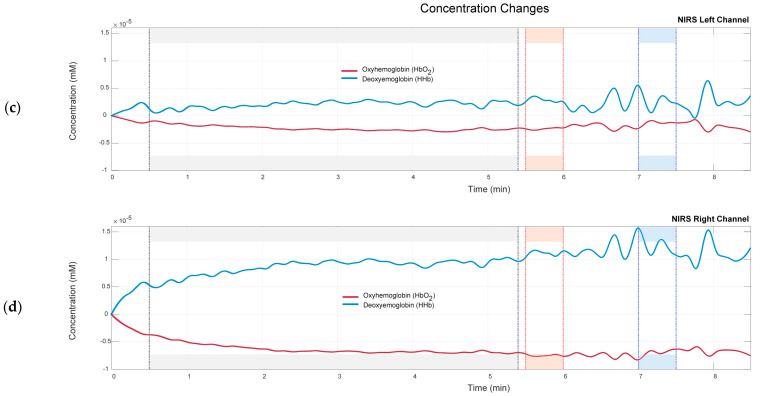
Concentrations of the chromophores for oxygenated (HbO_2_) and deoxygenated (HHb) hemoglobin for stroke patient 1. The gray area corresponds to the rest state, the light orange area corresponds to the right-hand movement, and the light blue area corresponds to the left-hand movement. (**a**) Oxygen saturation NIRS left channel. (**b**) Oxygen saturation NIRS right channel. (**c**) Concentration changes during rest and hand activation, NIRS left channel. (**d**) Concentration changes during rest and hand activation, NIRS right channel.

Finally, the concentrations of HbO_2_ and HHb are calculated using the equations and the algorithm described in Table 1, and the concentration graphs of both chromophores HbO_2_ and HHb are obtained. In Figure 5 and Figure 6, the oxygenated and deoxygenated hemoglobin found in the healthy subjects and stroke patients during rest and when performing a motor task is shown.

Descriptive statistics are performed on the healthy subjects’ data. Table 2 shows that the oxygen saturation levels expressed in percentage (%) between rest and left-hand movement for females, SpO_2_ = 97.49%, have a *p* = 0.016 significant value, meaning this value could differentiate between rest and a motor task. On the right side, during the motor task, with 93.10% of SpO_2_, there is a *p* = 0.018 significant value, differentiating for rest.

In the case of the male subjects, the differences in the SpO_2_ levels of rest and the left-hand movement (96.42%) are significant (*p* = 0.005), while in the rest and the right-hand movement (94.71%) they are not (*p* = 0.088).

Table 3 presents the concentrations of deoxyhemoglobin when comparing motor tasks to rest. In the case of the healthy female subjects, the mean concentration value is −1.88 ± 0.70 mM for rest, 0.16 ± 2.0 mM for the right-hand motor task, and 0.04 ± 1.84 mM for the left-hand motor task.

For the healthy male subjects, the mean concentration value is 1.94 ± 1.71 mM for rest, −2.74 ± 4.16 mM for the right-hand motor task, and −0.88 ± 1.17 mM for the left-hand motor task.

The only statistically significant hypothesis when comparing rest and motor tasks involved the male healthy subjects performing left-hand movement, with a *p*-value of 0.008.

## 4. Discussion

In this work, we used a sensor that is composed of photodiodes functioning as emitter optodes; these sensors have a high signal–noise ratio and a ready-to-use form factor. The sensor is used to calculate changes in the HbO_2_ and HHb chromophore concentration through a modification of the Beer–Lambert Law. The literature reports the use of this law to monitor tissue oxygen status, the most common application being for monitoring during cardiac surgery, intensive care units, and neonates [17]. Only a few works are related to assessing the effects of physiotherapy in chronic stroke patients [19]. As indicated by Wei-Liang et al., the sources of physiological noise present in fNIRS signals include heart rate, blood pressure fluctuations, respiratory rate, and scalp blood flow [20].

Oximetry from fNIRS signals assesses changes in oxygen levels. In the fNIRS signals, the changes in concentration in both oxygenated hemoglobin and deoxygenated hemoglobin can be identified, giving additional insights, as mentioned in the literature [21]. According to our results, NIRS sensors allow non-invasive monitoring along the rehabilitation therapy process of the changes in cerebral oxygen saturation in two regions with precise temporal resolution (matching the cardiac cycle), seeking to associate these changes with cerebral blood flow and the metabolism of stroke patients. The fNIRS signals could be valuable for tracking oxygen levels during the treatment of acute ischemic stroke [22].

One of the challenges that we noticed is that if we do not consider the similarity of the values in the current intensity signal of the reflected light between the left and right NIRS sensors, variations of over 15% occur in the calculations of oxygen saturation that somewhat echoes in the values of the changes in the concentrations of oxyhemoglobin and deoxyhemoglobin.

To avoid these variations and achieve similarity between the signals, we found that small movements of the sensors must be performed. If the sensor is moved towards the C_z_ position, the current values of the reflected light increase; on the contrary, if the displacement is towards the Nasion, the current values decrease. In the values recorded in the sample of healthy subjects, these range from a minimum of 0.010 μA to a maximum value of 0.090 μA in reflected red light.

The artifact that must also be considered in the adjustments is the high-frequency component; it is the most evident when adjusting the position of the NIRS, the position where its greatest attenuation was achieved, resulting in an improvement in the quality of the fNIRS signal, especially in the reflected red light which was 10% between F_p_ and F_z_.

In the sample of the healthy subjects, in the case of women, it is observed that it could apparently be associated with dominance; all the participants were right-handed, where the oxygen consumption reported by the left NIRS sensor is higher, 95.13 ± 1.46% left vs. 91.12 ± 1.09% right, at rest and in movement 97.49 ± 0.79% left vs. 93.10 ± 1.03%.

Regarding men, the mean values of oxygen consumption are not so different, achieving differentiation only on the left side where SpO_2_ is 93.42 ± 0.69% at rest vs. 96.42 ± 1.60% during the motor task. This could be attributed to anatomical differences between the skulls of women and men. Furthermore, in future works, these signals could be investigated to detect which side is involved in performing the movement.

During the 5 min rest stage, around minute 3, we see a drop in SpO_2_ levels, as shown in Figure 3. This decrease in oxygenation values is likely due to the subjects’ relaxing during rest. The increases in saturation toward the end may relate to the brain’s preparation when being prompted with a motor task a few seconds before. It is important to note that there is a delay in the body’s response to oxygen consumption. While the sensor could maybe differentiate between rest and motor tasks, changes in oxygen saturation take time to show.

In the healthy subjects, the concentrations of oxyhemoglobin and deoxyhemoglobin fluctuate at rest. Before the motor tasks, we see an increase in oxyhemoglobin concentration, as noted in Figure 4. Both sensors report similar concentration levels. When performing the measurements with the fNIRS sensor, we observed a latency related to the changes in task demand. This means that when the subject goes from the rest stage to the motor task, the changes in SpO_2_, oxyhemoglobin, and deoxyhemoglobin concentrations are noticeable a few seconds after the task starts.

All these changes must be further investigated in a future pilot study with a larger set of healthy subjects to help establish the reference values and/or behaviors expected from these innovative signals.

In the evaluated patients, the records of oxygen saturation indicate a decrease that may be linked to the affected side of the brain, as illustrated in Figure 5b (patient 1, affected brain side). Conversely, Figure 5a (patient 1, healthy brain side) shows a decrease during the rest stage, which could be associated with relaxation. Faced with the demand of the motor task, the oxygen saturation values increase.

Functional near-infrared spectroscopy is a valuable tool for monitoring patients who have suffered a stroke due to its ability to provide real-time, non-invasive insights into cerebral oxygenation and hemodynamics. fNIRS uses near-infrared light to measure changes in the oxygenated and deoxygenated hemoglobin concentrations in the brain, offering direct information about blood flow and oxygen levels in the affected regions. This technology is particularly useful for stroke patients because it allows clinicians to assess brain perfusion and detect areas with compromised blood flow, which is essential for understanding the extent of brain injury and guiding rehabilitation strategies. Additionally, fNIRS can be applied in bedside monitoring, making it accessible for continuous assessment during the acute phase and throughout recovery, ultimately contributing to personalized treatment and improved outcomes.

This study has certain limitations, including a small sample size of both healthy participants and post-stroke patients. Consequently, the findings should be validated with larger studies that could explore additional variables, such as incorporating other hand movements and extending rest periods. Such adjustments might help determine, for example, whether the observed increase in tissue oxygenation precedes the movement itself, potentially triggered by the anticipation of the instruction. The latency observed—referring to the time it takes for the signal to return to its baseline after a movement—warrants further investigation. Incorporating a cognitive task into the protocol could provide insights into how oxygenation patterns adapt to different demands. Heart rate can also be derived from the same NIRS signal, enabling the calculation of heart rate variability (HRV) indices during rest and movement phases. This would help explore the responses of the sympathetic and parasympathetic branches of the central nervous system and how these changes correlate with fluctuations in brain tissue oxygenation.

In future work, the continuous characterization of the NIRS sensors will have significant potential in post-stroke treatment evaluation. A key area is the development of algorithms that consider individual differences in tissue composition and scattering properties, which could affect measurement accuracy. Another promising path is the development of adaptive calibration models that can adjust to different patient conditions, such as changes in blood pressure, heart rate variability, and dynamic brain states, thus ensuring the reliable monitoring of hemodynamic responses to therapy. Furthermore, integrating fNIRS data with other neuroimaging modalities, such as fMRI or EEG, could deepen the understanding of neurovascular coupling and improve the monitoring of recovery processes. Finally, investigating machine learning approaches to analyze NIRS data can facilitate the predictive modeling of patients’ responses to rehabilitation, guiding personalized treatment plans and potentially improving long-term outcomes for stroke survivors.

## 5. Conclusions

Oxygen saturation levels for females show a significant difference between resting state and motor tasks (*p* = 0.016) for left-hand movement and right-side movement (*p* = 0.018). For males, oxygen saturation corresponding to the left hand at rest significantly differs from motor task values (*p* = 0.005), while for the right hand, it does not show significance (*p* = 0.088). Similar results were found by Chang et al. [23], where a significant increase in oxygenated hemoglobin was observed in the prefrontal cortex during passive movements. Other authors have tested these increments for lower limb protocols in post-stroke patients and other pathologies as well [24].

From this work, it is seen that the NIRS sensor is not complex to use when one has the right guidelines. This paper describes the methodology to position the NIRS sensors, process the signals, and translate the mathematical model into a practical algorithm to calculate oxygen saturation and the oxyhemoglobin and deoxyhemoglobin concentrations from fNIRS raw signals. This sensor is non-invasive, easy to place, and well-accepted by the patient. The protocol for signal acquisition is easy to perform by healthy subjects and stroke survivor patients. This study demonstrates that it is feasible to evaluate rehabilitation therapy using NIRS sensors.

## Figures and Tables

**Table 2:** Normality test and statistical analysis for oxygen saturation values, healthy subjects.

Oxygen Saturation SpO_2_ (%)							
	**Condition**	Left Hand			Right Hand		
Females	Rest	95.12	Normality Test (*p* = 0.014)	^1^ Ha μ Rest ≠ μ Movement * p = 0.016	91.79	Normality Test (*p* = 0.362)	^1^ Ha μ Rest ≠ μ Movement ** p = 0.018
		96.31			91.12		
		95.54			89.32		
		92.65			91.22		
		96.02			92.13		
	Mean ± SD	95.13 ± 1.46			91.12 ± 1.09		
	Movement	97.77			93.62		
		97.90			92.39		
		97.87			91.72		
		96.07			94.29		
		97.82			93.48		
	Mean ± SD	97.49 ± 0.79			93.10 ± 1.03		
	**Condition**	**Left Hand**			**Right Hand**		
Males	Rest	94.38	Normality Test (*p* = 0.551)	^1^ Ha μ Rest ≠ μ Movement ** p = 0.005	93.16	Normality Test (*p* = 0.201)	^1^ Ha μ Rest ≠ μ Movement ** p = 0.088
		93.05			95.10		
		92.88			91.52		
		93.91			92.06		
		92.86			91.50		
	Mean ± SD	93.42 ± 0.69			92.67 ± 1.52		
	Movement	97.90			96.43		
		97.50			96.68		
		94.26			92.90		
		97.25			94.40		
		95.20			93.12		
	Mean ± SD	96.42 ± 1.60			94.71 ± 1.78		

For the normality assessment, the Shapiro-Wilk test was performed for all cases. ^1^ significant difference in oxygen saturation levels between rest and hand movement. * Mann-Whitney U test. ** Student’s T test.

**Table 3:** Normality test and statistical analysis for the concentration of deoxyhemoglobin values, healthy subjects.

Concentration Changes (mM) Deoxyhemoglobin Concentration (HHb)							
	Condition	Left Hand			Right Hand		
Females	Rest	−2.8	Normality Test (*p* = 0.661)	Ha μ Rest ≠ μ Movement * p = 0.075	−0.3	Normality Test (*p* = 0.934)	Ha μ Rest ≠ μ Movement * p = 1.0
		−1.2			−1.8		
		−1.3			0.3		
		−2.4			4.2		
		−1.7			1.8		
	Mean ± SD	−1.88 ± 0.70			0.84 ± 2.28		
	Movement	−0.5			0.5		
		0.9			−3.1		
		−1.8			0.3		
		2.8			2.4		
		−1.2			0.7		
	Mean ± SD	0.04 ± 1.84			0.16 ± 2.0		
	**Condition**	**Left Hand**			**Right Hand**		
Males	Rest	2.2	Normality Test (*p* = 0.759)	^1^ Ha μ Rest ≠ μ Movement * p = 0.008	−1.4	Normality Test (*p* = 0.428)	Ha μ Rest ≠ μ Movement * p = 0.548
		4.8			−0.7		
		0.8			3.6		
		0.6			−7.1		
		1.3			−3.2		
	Mean ± SD	1.94 ± 1.71			−1.76 ± 3.89		
	Movement	−0.3			−1.8		
		0.3			−2.1		
		−2.8			3.2		
		−0.9			−8.0		
		−0.7			−5.0		
	Mean ± SD	−0.88 ± 1.17			−2.74 ± 4.16		

For the normality assessment, the Shapiro-Wilk test was performed for all cases. ^1^ significant difference in deoxyhemoglobin concentration values between rest and hand movement. * Mann-Whitney U test

## Data Availability

Data are available upon request through the IRB Committee.

## Abbreviations

AC	Alternating Current
AR	red/ired Absorbance Ratio
CT	Computed Tomography
DC	Direct Current
DPF	Differential Path Factor
DW	Discrete Wavelet Transform
fNIRS	functional Near-Infrared Spectroscopy
HbO_2_	Oxygenated Hemoglobin
HHb	Deoxygenated Hemoglobin
ired	Infrared Signals
MRI	Magnetic Resonance Imaging
NIRS	Near-Infrared Spectroscopy
PET	Positron Emission Tomography
red	Red Signals
SpO_2_	Oxygen Saturation
VPP	Peak-to-Peak Value
